# Multimodal salvage therapy for locally advanced esophageal cancer recurrence after failed post-ESD surveillance: a case report

**DOI:** 10.3389/fonc.2026.1852836

**Published:** 2026-07-10

**Authors:** Mengzhi Cheng, Henghui Bian, Xuhua Cai, Jianbin Zhang

**Affiliations:** 1 Department of Thoracic Surgery, Huzhou Central Hospital, Fifth School of Clinical Medicine of Zhejiang Chinese Medical University, Huzhou, China; 2 Department of Thoracic Surgery, Huzhou Central Hospital, Affiliated Central Hospital of Huzhou University, Huzhou, China; 3 Department of Gastroenterology, Huzhou Central Hospital, Fifth School of Clinical Medicine of Zhejiang Chinese Medical University, Huzhou, China; 4 Department of Gastroenterology, Huzhou Central Hospital, Affiliated Central Hospital of Huzhou University, Huzhou, China

**Keywords:** endoscopic submucosal dissection, esophageal squamous cell carcinoma, neoadjuvant chemoimmunotherapy, salvage esophagectomy, surveillance failure

## Abstract

Endoscopic submucosal dissection (ESD) is the standard curative treatment for early esophageal squamous cell carcinoma (ESCC). However, its long-term success critically depends on rigorous postoperative surveillance to detect recurrence at a curable stage. We report a 66-year-old man who underwent ESD that met the expanded criteria for early ESCC (pT1a-MM with negative margins and no lymphovascular invasion) but was subsequently lost to follow-up. He represented 17 months later with dysphagia and was diagnosed with locally advanced recurrence (cT3N2M0). Following multidisciplinary team assessment, he received two cycles of neoadjuvant chemoimmunotherapy (albumin-bound paclitaxel, cisplatin, and camrelizumab), achieving marked regression of both the primary tumor and regional lymph nodes. He then underwent radical transthoracic esophagectomy, with final pathology confirming ypT2N0M0 disease. After two cycles of adjuvant chemoimmunotherapy, he entered intensive surveillance and remained disease-free at the last follow-up in September 2025. This case delivers two essential lessons: loss to follow−up after curative ESD precludes early detection of recurrence, potentially resulting in rapid progression to life−threatening disease; yet even in this setting, a timely multimodal salvage strategy can still offer the possibility of long−term survival.

## Introduction

Endoscopic submucosal dissection (ESD) is a well-established curative treatment for early esophageal squamous cell carcinoma (ESCC), with long-term outcomes comparable to surgery (1). Its success, however, depends on two essential components: a histologically complete (R0) resection and, equally importantly, the patient’s adherence to structured postoperative surveillance. Previous studies have demonstrated that post−ESD surveillance may detect local or metachronous recurrence at a stage that remains amenable to repeat endoscopic therapy (2, 3). A critical vulnerability in this paradigm is patient disengagement. When surveillance fails, the opportunity for early detection and intervention is lost. In such cases, a potentially manageable local recurrence may progress, often rapidly, to locally advanced, node-positive disease. We present this case not only to illustrate a successful multimodal salvage but, more importantly, to highlight the catastrophic consequences of surveillance failure and the imperative for robust follow-up systems.

## Case presentation

In December 2021, a 66−year−old man with hypertension and type 2 diabetes underwent routine endoscopy, which revealed a lesion in the distal esophagus (**Figure 1A**), and biopsy confirmed high−grade intraepithelial neoplasia with focal squamous cell carcinoma. Staging with endoscopic ultrasonography and contrast−enhanced CT confirmed cT1aN0M0 ESCC. The patient therefore underwent curative ESD (**Figure 1B**). Histopathological examination of the resected specimen (2.0 × 1.9 × 0.5 cm) confirmed a moderately differentiated squamous cell carcinoma (1.7 × 1.5 cm) invading the muscularis mucosae (**Figure 1D**). All resection margins were negative. The deep (basal) margin measured 0.05 cm, and there was no lymphovascular invasion. Resection fulfilled the technical criteria for R0 resection; however, per the Japan Gastroenterological Endoscopy Society (JGES) 2020 guidelines (1), pT1a-MM invasion falls into the expanded indication category, which recommends additional treatment or extremely rigorous surveillance. Upon discharge, he was advised to adhere to a standard post−ESD surveillance protocol.

**Figure 1:**
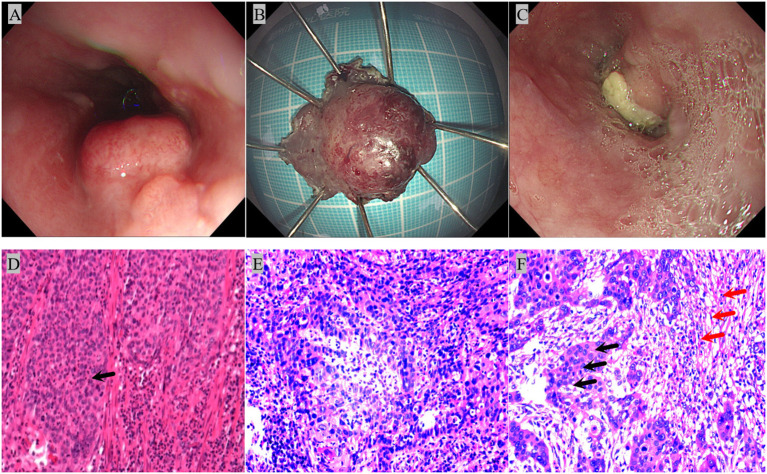
Endoscopic and histopathological findings. **(A)** Initial endoscopic view showing a lesion in the distal esophagus before endoscopic submucosal dissection (ESD). **(B)** Resected ESD specimen measuring 2.0 × 1.9 × 0.5 cm. **(C)** Endoscopy at the time of recurrence (17 months after ESD) revealing an ulcerated mass at the previous ESD site. **(D)** Histopathology of the ESD specimen showing a moderately differentiated squamous cell carcinoma invading the muscularis mucosae (haematoxylin and eosin [H&E], ×100). **(E)** Biopsy of the recurrent mass confirming recurrent squamous cell carcinoma (H&E, ×100). **(F)** Histopathology of the resected specimen after neoadjuvant chemoimmunotherapy showing residual moderately differentiated squamous cell carcinoma with extensive treatment−related fibrosis (H&E, ×100).

Critically, the patient was lost to follow−up. He represented 17 months later, in May 2023, with progressive dysphagia. Endoscopy revealed an ulcerated mass at the previous ESD site (**Figure 1C**). Biopsy confirmed recurrent squamous cell carcinoma (**Figure 1E**). Positron emission tomography-computed tomography (PET-CT) was declined by the patient due to financial constraints, and endoscopic ultrasonography (EUS) was precluded by severe esophageal stenosis. Contrast−enhanced CT showed eccentric wall thickening with probable transmural invasion and multiple enlarged regional lymph nodes (**Figures 2A–D**). No distant metastases were found. Based on these findings, clinical stage was now cT3N2M0 (AJCC 8th edition) (4) — a dramatic progression attributable to the surveillance lapse.

**Figure 2:**
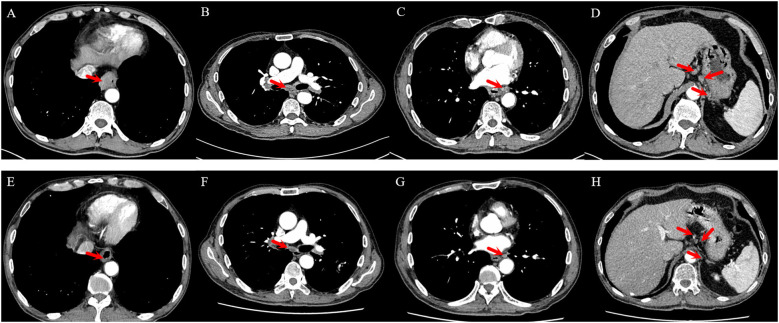
Contrast-enhanced CT images before and after neoadjuvant chemoimmunotherapy. **(A–D)** CT images at the time of recurrence (May 2023), showing eccentric wall thickening of the distal esophagus (arrow, **(A)**) and multiple enlarged regional lymph nodes (arrows, **(B–D)**), consistent with cT3N2M0 disease. **(E–H)** CT images after two cycles of neoadjuvant chemoimmunotherapy (July 2023), demonstrating marked regression of both the primary tumour (arrow, **(E)**) and the involved lymph nodes (arrows, **(F–H)**).

Following multidisciplinary team (MDT) discussion, a consensus was reached to pursue a comprehensive salvage strategy comprising neoadjuvant therapy, radical surgery, and adjuvant therapy, with the aim of maximizing long-term outcomes. After providing written informed consent, the patient received two cycles of neoadjuvant chemoimmunotherapy (albumin-bound paclitaxel 400 mg on day 1, cisplatin 40 mg on days 1–3, and camrelizumab 200 mg on day 1 of a 21-day cycle) from May to June 2023. Restaging after two cycles revealed marked radiological regression of both primary tumour and lymph nodes (**Figures 2E–H**). In July 2023, he underwent radical transthoracic esophagectomy via a right thoracic approach (Ivor-Lewis procedure) with two-field lymphadenectomy (thoracic and abdominal nodal stations). Final pathology showed a residual moderately differentiated squamous cell carcinoma (1.8 × 1.5 cm) infiltrating the deep muscularis propria, with extensive treatment-related fibrosis replacing the majority of the tumor bed, with only scattered residual tumor cell nests identified (**Figure 1F**). All 31 resected lymph nodes and margins were negative. Postoperative pathological stage was ypT2N0M0. His postoperative recovery was uneventful, and he was discharged on postoperative day 10. He received two cycles of adjuvant chemoimmunotherapy with the same regimen and then entered intensive surveillance. At the latest follow−up in September 2025, he remains disease−free. The treatment timeline is summarised in **Figure 3**.

**Figure 3:**
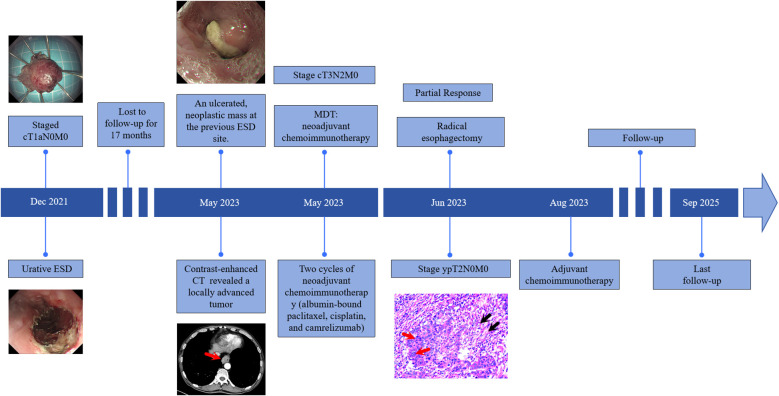
Treatment timeline. Schematic timeline summarising the patient’s clinical course from initial ESD in December 2021 to the last follow-up in September 2025. Key events include ESD, recurrence diagnosis, neoadjuvant chemoimmunotherapy, radical surgery, adjuvant therapy, and surveillance.

## Discussion

This case delivers a stark warning: the curative intent of ESD for early esophageal cancer is contingent not only on technical success but, equally importantly, on rigorous postoperative surveillance. Our patient’s trajectory—from a curative R0 resection to life-threatening locally advanced recurrence—illustrates the consequences when this surveillance fails.

Post−ESD surveillance is designed to detect local or metachronous recurrence within the first 18–24 months after resection. The risk of recurrence during this period is reported to be 2–6% (5); for patients who develop local recurrence during this window, repeat ESD is an established salvage treatment (3). In well−followed patients, lymph node metastasis is exceptionally rare, with reported rates ranging from 0% for tumours confined to the epithelium or lamina propria to 5.6% for those invading the muscularis mucosae (2). In stark contrast, our case exemplifies a critical deviation from the expected course following curative ESD, directly attributable to the failure of postoperative surveillance. The 17−month period of unmonitored follow−up resulted in progression of an otherwise manageable local recurrence to symptomatic locally advanced disease. This rapid progression serves as a stark reminder that local recurrence following ESD can progress aggressively. Furthermore, the recurrent tumour at the prior ESD site exhibited an eccentric growth pattern (**Figure 2A**), which may have postponed the onset of obstructive dysphagia, ultimately resulting in clinical presentation at an advanced, locally invasive stage. Thus, this surveillance failure constitutes a “double jeopardy” scenario — delayed detection coupled with the potential for accelerated progression.

This case underscores a fundamental vulnerability in the organ-preservation paradigm: its dependence on sustained patient adherence, a factor often beyond the physician’s direct control. The passive follow−up model—relying on patients to initiate scheduled surveillance—proved insufficient in this instance. This observation is supported by a growing body of evidence demonstrating that structured recall systems, including registry−based reminders and dedicated nurse coordinators, can significantly improve adherence to post−treatment surveillance protocols (6, 7). While the optimal approach requires further investigation, transitioning from passive to active follow−up systems should be considered to mitigate the risk of loss to follow−up.

The salvage therapy for this locally advanced recurrence followed the same principle as for *de novo* advanced ESCC: neoadjuvant systemic therapy followed by radical resection. We selected neoadjuvant chemoimmunotherapy based on contemporary evidence demonstrating its superiority over chemotherapy alone in both metastatic and locally advanced settings (8). The favourable pathological response (ypT2N0) confirmed successful downstaging, and R0 resection was achieved. Adjuvant chemoimmunotherapy was administered to mitigate residual risk, an approach supported by trials demonstrating benefit in patients with residual disease after neoadjuvant treatment (9).

Collectively, these observations highlight two essential messages for clinical practice. First, post-ESD surveillance is the keystone of curative intent. Strengthening follow−up through patient education and active recall systems—such as registry−based reminders and nurse coordinators—deserves equal priority with technical training in ESD. Second, when prevention fails, a timely MDT−driven multimodal strategy integrating perioperative chemoimmunotherapy and radical surgery can still achieve long−term disease control. Clinicians should maintain a high index of suspicion for dysphagia after ESD and pursue immediate imaging and endoscopy; if locally advanced recurrence is confirmed, urgent referral for multimodal salvage therapy comprising perioperative chemoimmunotherapy and radical surgery is essential.

Several limitations should be acknowledged. First, although the recurrent mass was located at the prior ESD scar—the defining criterion for local recurrence per the Japan Esophageal Society (JES) guidelines—the possibility of a metachronous primary tumor cannot be excluded without genetic analysis or supportive immunohistochemical evidence. Second, routine Tumor Regression Grade (TRG) scoring was unavailable at our institution in 2023; we therefore reported descriptive findings of treatment-induced fibrosis. Despite these limitations, this case highlights two key messages: the critical importance of rigorous post-ESD surveillance, and the potential for timely multimodal salvage therapy to achieve long-term disease control even after surveillance failure.

## Conclusions

This case serves as a cautionary tale: loss to follow-up after curative ESD for early esophageal cancer can have catastrophic consequences. Clinicians must prioritise robust surveillance systems alongside technical excellence in endoscopic resection. When surveillance fails, however, timely multimodal salvage therapy — including chemoimmunotherapy and radical surgery — can still achieve a favorable outcome.

## Data Availability

The original contributions presented in the study are included in the article/**Supplementary Material**, further inquiries can be directed to the corresponding author/s.
