# Supplementary material for: Multimodal salvage therapy for locally advanced esophageal cancer recurrence after failed post-ESD surveillance: a case report
Source: Front Oncol. 2026 Jul 10;16:1852836. doi: 10.3389/fonc.2026.1852836 (PMC13397105; doi:10.3389/fonc.2026.1852836)
Supplement: Supplementary file 1 [file Table1.docx]

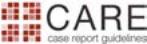

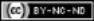

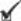
**CARE Checklist of information to include when writing a case report**

| **Topic** | **Item No** | **Checklist item description** | **Reported on Page Number/Line**  **Number** | **Reported on**  **Section/Paragraph** |  |
| --- | --- | --- | --- | --- | --- |
| Title | 1 | The diagnosis or intervention of primary focus followed by the words “case report” | Page 1/Line 1-2 | Title |  |
| Key Words | 2 | 2 to 5 key words that identify diagnoses or interventions in this case report, including "case report" | Page 2/Line 44-45 | Keywords |  |
| Abstract  (no references) | 3a | Background: why the case report is unique and what it adds to existing literature? | Page 2/Line 27-34 | Abstract |  |
|  | 3b | Main symptoms and/or important clinical findings | Page 2/Line 32-34 | Abstract |  |
|  | 3c | The main diagnoses, therapeutic interventions, and outcomes | Page 2/Line 34-40 | Abstract |  |
|  | 3d | Conclusion—What is the main “take-away” lesson(s) from this case? | Page 1/Line 40-43 | Abstract |  |
| Introduction | 4 | One or two paragraphs summarizing why this case is unique **(may include references)** | Page 3/Line 55-68 | Introduction |  |
| Patient Information | 5a | De-identified patient specific information | Page 3/Line 69-73 | Case Presentation |  |
|  | 5b | Primary concerns and symptoms of the patient | Page 3/Line 70-71;81-82 | Case Presentation |  |
|  | 5c | Medical, family, and psycho-social history including relevant genetic information | Page 3/Line 70-71 | Case Presentation |  |
|  | 5d | Relevant past interventions with outcomes | Page 3/Line 73-80 | Case Presentation |  |
| Clinical Findings | 6 | Describe significant physical examination (PE) and important clinical findings | Page 3/Line 81-82 | Case Presentation |  |
| Timeline | 7 | Historical and current information from this episode of care organized as a timeline | Page 4/Line 103 | Case Presentation |  |
| Diagnostic Assessment | 8a | Diagnostic testing (such as PE, laboratory testing, imaging, surveys). | Page 3-4/Line 82-86 | Case Presentation |  |
|  | 8b | Diagnostic challenges (such as access to testing, financial, or cultural) | N/A | N/A |  |
|  | 8c | Diagnosis (including other diagnoses considered) | N/A | N/A |  |
|  | 8d | Prognosis (such as staging in oncology) where applicable | Page 4/Line 86-87 | Case Presentation |  |
| Therapeutic Intervention | 9a | Types of therapeutic intervention (such as pharmacologic, surgical, preventive, self-care) | Page 4/Line 88-90 | Case Presentation |  |
|  | 9b | Administration of therapeutic intervention (such as dosage, strength, duration) | Page 4/Line 90-93 | Case Presentation |  |
|  | 9c | Changes in therapeutic intervention (with rationale) | Page 4/Line 93-99 | Case Presentation |  |

| Follow-up and Outcomes | 10a | Clinician and patient-assessed outcomes (if available) | Page 4/Line 99-102 | Case Presentation |
| --- | --- | --- | --- | --- |
|  | 10b | Important follow-up diagnostic and other test results | Page 4/Line 99-102 | Case Presentation |
|  | 10c | Intervention adherence and tolerability (How was this assessed?) | Page 4/Line 99-102 | Case Presentation |
|  | 10d | Adverse and unanticipated events | Page 4/Line 102 | Case Presentation |
| Discussion | 11a | A scientific discussion of the strengths AND limitations associated with this case report | Page 4/Line 104-109 | Discussion |
|  | 11b | Discussion of the relevant medical literature **with references** | Page 4-5/Line 110-144 | Discussion |
|  | 11c | The scientific rationale for any conclusions (including assessment of possible causes) | Page 4-5/Line 110-144 | Discussion |
|  | 11d | The primary “take-away” lessons of this case report (without references) in a one paragraph conclusion | Page 5-6/Line 145-163 | Discussion |
| Patient Perspective | 12 | The patient should share their perspective in one to two paragraphs on the treatment(s) they received | N/A | N/A |
| Informed Consent | 13 | Did the patient give informed consent? Please provide if requested | **Yes** 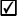 | **No** 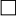 |

| Please leave this space alone as it will be supplemented by the editorial office when needed. |
| --- |

2-2 Updated on February 20, 2026
